# Multiscale permutation Rényi entropy and its application for EEG signals

**DOI:** 10.1371/journal.pone.0202558

**Published:** 2018-09-04

**Authors:** Yinghuang Yin, Kehui Sun, Shaobo He

**Affiliations:** 1 School of Physics and Electronics, Central South University, Changsha, P.R.China; 2 School of Computer Science and Technology, Hunan University of Arts and Science, Changde, P.R.China; University of Adelaide, AUSTRALIA

## Abstract

There is considerable interest in analyzing the complexity of electroencephalography (EEG) signals. However, some traditional complexity measure algorithms only quantify the complexities of signals, but cannot discriminate different signals very well. To analyze the complexity of epileptic EEG signals better, a new multiscale permutation Rényi entropy (MPEr) algorithm is proposed. In this algorithm, the coarse-grained procedure is introduced by using weighting-averaging method, and the weighted factors are determined by analyzing nonlinear signals. We apply the new algorithm to analyze epileptic EEG signals. The experimental results show that MPEr algorithm has good performance for discriminating different EEG signals. Compared with permutation Rényi entropy (PEr) and multiscale permutation entropy (MPE), MPEr distinguishes different EEG signals successfully. The proposed MPEr algorithm is effective and has good applications prospects in EEG signals analysis.

## Introduction

Electroencephalography (EEG) is an electrophysiological monitoring method to measure the voltage fluctuations resulting from ionic current within the neurons of the brain. EEG signals can reflect the electrical activities of the neurons directly, and are closely related to health [1–3]. Some approaches about EEG classification are proposed, such as neural networks methods [4, 5], complex networks based on phase lag index (PLI) [6] and some entropy-based measures [7, 8], such as approximate entropy (ApEn) [9]. However, an EEG signal may be mixed with electromyography (EMG) signal and electrooculogram (EOG) signal, and it is difficult for people to extract the diagnostic features from the interfered signal. So people put forward some new EEG signal processing algorithms [10, 11]. At present, extracting useful information from EEG signals by using complexity methods becomes a hot topic [12–15]. So far, several entropy-based algorithms have been proposed, such as Lempel-Ziv algorithm [16], approximate entropy (ApEn) [17], fuzzy entropy (FuzzyEn) [18], sample entropy (SampEn) [19]. However, some of them cannot measure the complexity of EEG signals reliably [20–22]. In 2013, Zhao et al. used Rényi permutation entropy [23] to analyze time series, and soon Nadia Mammone et al. proposed permutation Rényi entropy (PEr) [24] based on permutation entropy (PE) [25], and it was applied to analyze childhood absence epilepsy EEG signals successfully [26]. It achieved favorable discriminating effectiveness between interictal states and ictal states [27, 28] in absence seizure EEG signals, but it only can discriminate two kinds of EEG signals. To analyze the EEG signals better, some modified methods are proposed, including multiscale entropy (MSE) [29], multivariate permutation entropy (MvPE) [30] and modified multiscale sample entropy (MMSE) [31]. The main modifications of those algorithms lie in the coarse-grained procedure, including average method or moving-averaging method. For average method, the data size is reduced after the original series is divided by the scale factor [32], and it leads to some information loss and inaccurate measurement [33–36]. Moving-averaging method can almost get the same data size, which keeps more complete information, but it has some defects in extracting effective signals and filtering interference signals. Apart from the coarse-grained methods mentioned above, some novel multiscale entropy are proposed to analyze time series. For example, Zunino et al. [37] utilized multiscale symbolic information-theory approach to characterize multiscaled time series, and Zunino et al. [38] identified the hidden temporal correlations in time series by using permutation min-entropy. So it is an interesting and challenging task to design a complexity measure algorithm to distinguish different EEG signals further.

In this paper, we proposed a new complexity measure algorithm, named multiscale permutation Rényi entropy (MPEr), and applied it to analyze epileptic EEG signals. The rest of this paper is organized as follows. Firstly, MPEr is proposed, and its performance is analyzed and compared with PEr and MPE. Then, MPEr is applied to analyze the complexity of different epileptic EEG signals and the statistical analysis is conducted. Performance comparisons with other complexity measure algorithms are carried out. Finally, we summarize the results and indicate the future work.

## Materials and methods

### Different coarse-grained methods

**Fig 1:**
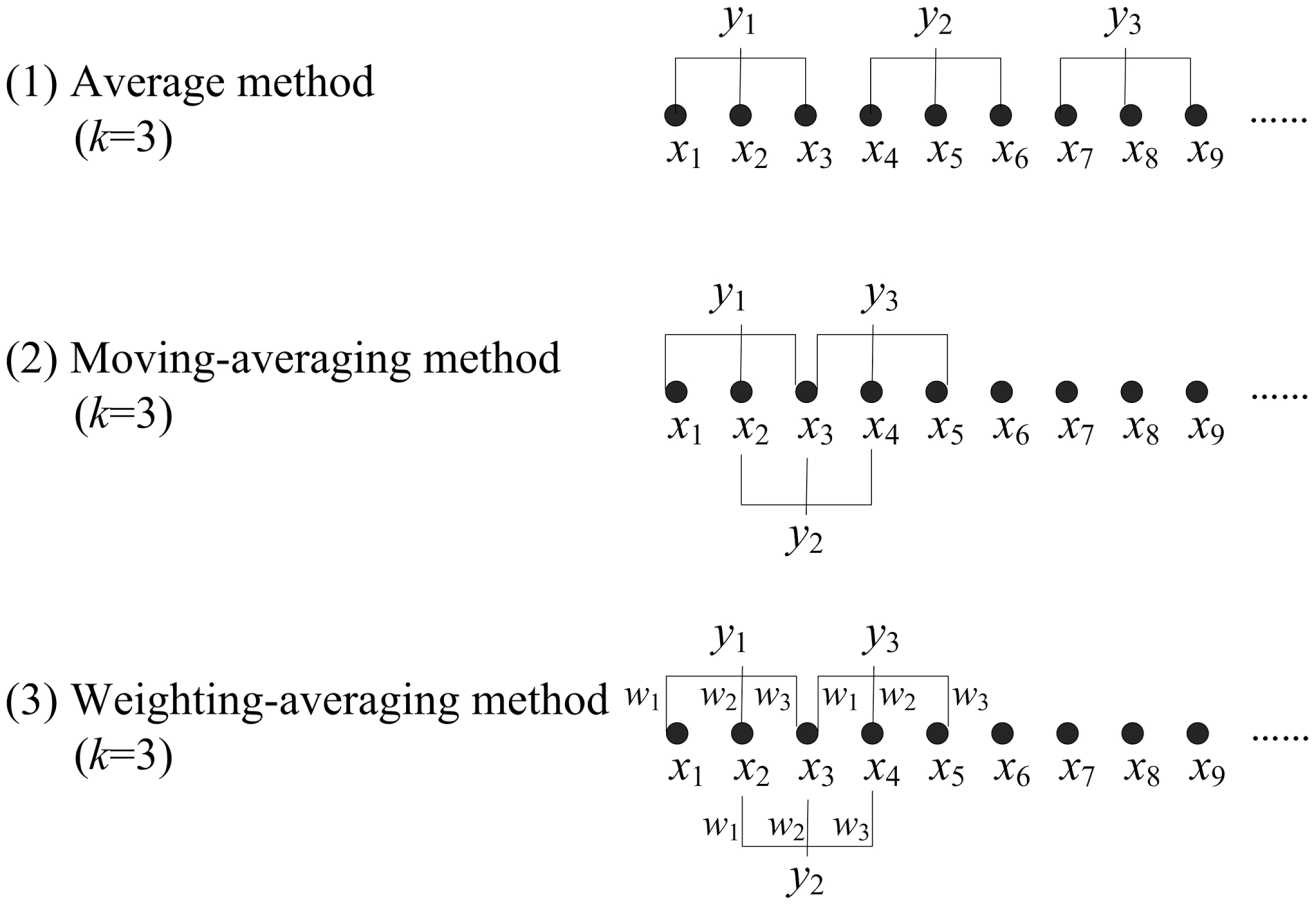
The reconstructed series *y*_*j*_ computed by different coarse-grained approaches (average, moving-averaging and weighting-averaging method). Note that *k* = 3 for each method.

1. Average method.
  For a given discrete time series *x*_1_, *x*_2_, …, *x*_*N*_, the average method is described as [29]
  $$\begin{array}{c} y_{j}^{\left(k\right)}=\frac{1}{k}\sum_{i=(j-1)k+1}^{jk}x_{i},1\leq j\leq N/k, \end{array}$$ (1)
  where *k* is the scale factor, and it determines the length of the reconstructed time series. $y_{j}^{\left(k\right)}$ is the coarse-grained time series, which is the same as the original time series when *k* = 1. The length of each coarse-grained time series is reduced to 1/*k* of the original series, so some useful information of the original time series may be lost.
2. Moving-averaging method.
  The moving-averaging method is defined by [39]
  $$\begin{array}{c} y_{j}^{\left(k\right)}=\frac{1}{k}\sum_{i=j}^{j+k-1}x_{i},1\leq j\leq N-k+1, \end{array}$$ (2)
  where *k* is the scale factor. $y_{j}^{\left(k\right)}$ is the coarse-grained time series, which is the same as the original time series when *k* = 1. According to Eq (2), the length of the coarse-grained series is *N* − *k* + 1, which is a little shorter than that of the original series. The moving-averaging method makes more full use of the information compared with the average method. However, it is difficult to filter interference signals.
3. Weighting-averaging method.
  Here, we introduce the weighted factors *w*_*n*_(*n* = 1, 2, …, *k*), and a new coarse-grained method named weighting-averaging method is defined by
  $$\begin{array}{c} y_{j}^{\left(k\right)}=\frac{\sum_{n=1}^{k}\sum_{i=j}^{j+k-1}w_{n}x_{i}}{\sum_{n=1}^{k}w_{n}},1\leq j\leq N-k+1, \end{array}$$ (3)
  where *w*_*n*_ is the weighted factor, and $y_{j}^{\left(k\right)}$ is the coarse-grained time series. *k* is the scale factor, which is equal to the number of the weighted factors. $y_{j}^{\left(k\right)}$ is the same as the original time series when *k* = 1. The larger weighted factors mean emphasis on the corresponding data. This method emphasizes the central information by lager central weighted factor. That is to say, for a given time series, the weighted factors increase first, and then decrease. It is the same with the moving-averaging method when *w*_*n*_ = 1, *n* = 1, 2, …, *k*. The different reconstructed series calculated by average, moving-averaging and weighting-averaging methods are visualized as shown in Fig 1.

### Multiscale permutation Rényi entropy

For a given finite time series *x*_*n*_, *n* = 1, 2, …, *N*, a restructuring matrix *X*_*r*_(*i*, *m*) is obtained by

$$\begin{array}{c} X_{r}\left(i,m\right)=[\begin{array}{cccc} x(1) & x(1+\tau ) & \cdots & x(1+(m-1)\tau )\\ x(2) & x(2+\tau ) & \cdots & x(2+(m-1)\tau )\\ \vdots & \vdots & & \vdots \\ x(l) & x(l+\tau ) & \cdots & x(l+(m-1)\tau )\\ \vdots & \vdots & & \vdots \\ x(i) & x(i+\tau ) & \cdots & x(i+(m-1)\tau ) \end{array}], \end{array}$$ (4)

where *l* = 1, 2, …, *i*. *m* is the embedding dimension and varies from 3 to 7. *τ* is the time lag, here, *τ* = 1 [24]. *i* = *N* − (*m* − 1)*τ*. For each row vector *X*_*l*_ of the restructuring matrix, we sort them in an ascending order *π* = (*r*_0_, *r*_1_, …, *r*_*m*−1_) to get the new row vectors $X_{r_{l}}$. The values of the new row vectors are described as

$$\begin{array}{c} x_{l+r_{0}}\leq x_{l+r_{1}}\leq \cdots \leq x_{l+r_{m-1}}. \end{array}$$ (5)

There are *m*! possible patterns. Assuming that *π*_*j*_ = *j*, *j* = 1, 2, …, *m*!, the arrangement patterns sequence *s*(*i*), *i* = 1, 2, …, *N* − *m* + 1 is obtained. According to the Bandt-Pompe probability distribution [25], the probability of each possible *π* is defined by

$$\begin{array}{c} p(\pi_{j})=\frac{\#\{s|i\leq N-m+1;s=j\}}{N-m+1}, \end{array}$$ (6)

where the symbol # denotes the number of the arrangement patterns.

According to Shannon entropy, the permutation Rényi entropy is defined by [23, 24]

$$\begin{array}{c} PEr(x,m,\alpha )=\frac{1}{1-\alpha }\log\sum_{i=1}^{m!}p{\left(\pi_{j}\right)}^{\alpha }, \end{array}$$ (7)

where *α* is a new parameter and it varies from 2 to 7.

To improve the performance of PEr further, the weighting-averaging method is introduced. For a given finite time series {*x*_*n*_, *n* = 1, 2, …, *N*}, the coarse-grained series $y_{j}^{\left(k\right)}$ is calculated according to Eq (3). Then we reconstruct $y_{j}^{\left(k\right)}$ with the embedding dimension *m* and the time lag *τ*, and acquire the probability of the arrangement patterns. So, the multiscale permutation Rényi entropy (MPEr) is defined by

$$\begin{array}{c} MPEr(x,m,\alpha ,w_{n})=\frac{1}{1-\alpha }\log\sum_{i=1}^{m!}p_{w_{n}}{\left(\pi_{j}\right)}^{\alpha }, \end{array}$$ (8)

where *w*_*n*_ is the weighted factor, and $p_{w_{n}}$ is the probability of arrangement patterns of the coarse-grained series. For MPEr, there are 3 parameters (*m*, *α*, *w*_*n*_). Once they are determined, they are suitable for different groups of subjects. Next, we will describe how to determine these parameters.

### Complexity analysis of the chaotic signals

Some medical signals have the similar dynamic characteristics as chaotic signals, so, we use chaotic signals to analyze the influence of parameters on the algorithm. Here, the two-dimensional (2D) SF-SIMM (sinusoidal feedback Sine ICMIC (iterative chaotic map with infinite collapse) modulation map) is considered [40, 41]

$$\left\{ \begin{array}{c} x(n+1)=\mu \sin[\omega y(n)]\sin[c/x(n)],\\ y(n+1)=\mu \sin[\omega x(n+1)]\sin[c/y(n)]. \end{array}\right.$$ (9)

**Fig 2:**
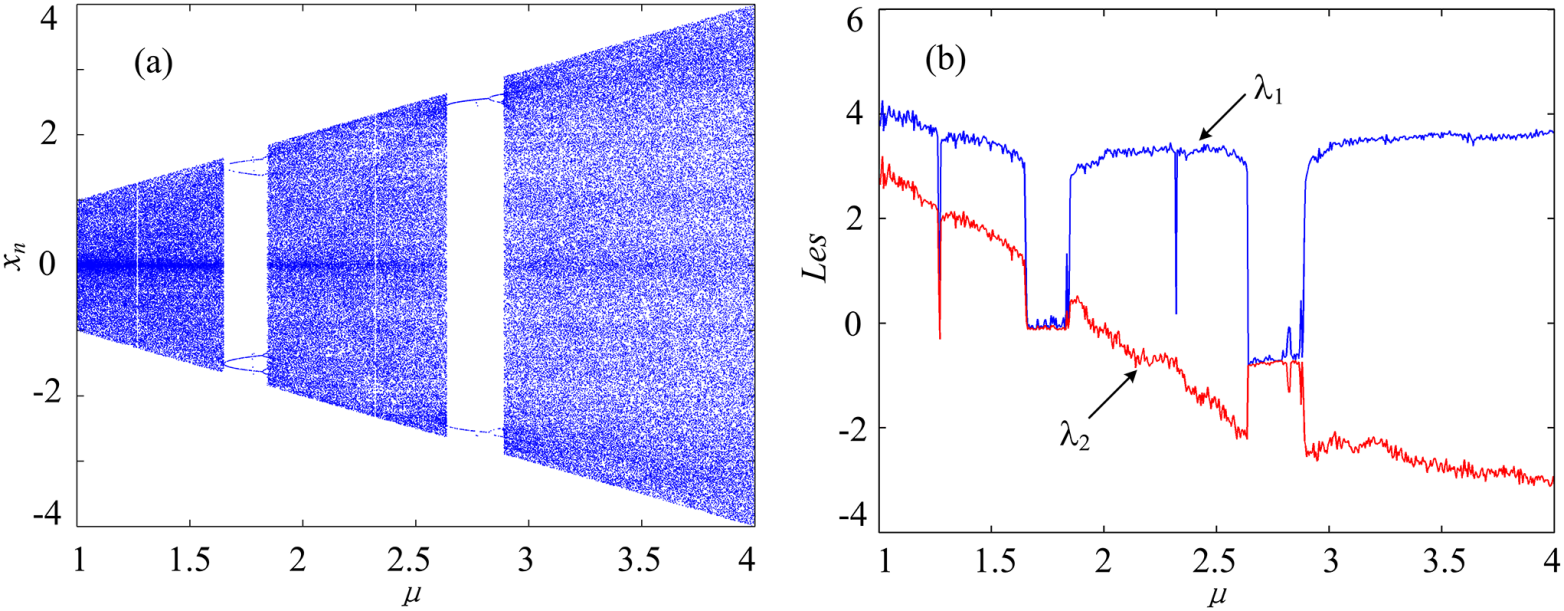
Dynamics of the 2D SF-SIMM by setting initial value (0.3, 0.4), iteration times *N* = 1000, system parameter *b* = 3 and *μ* varying from 1 to 4 with the step size of 0.01. (a) Bifurcation diagram. (b) Lyapunov exponent spectrum.

Setting initial value (*x*_0_, *y*_0_) = (0.3, 0.4), iteration times *N* = 1000, system parameter *b* = 3 and *μ* varying from 1 to 4 with the step size of 0.01, the bifurcation diagram and Lyapunov exponent spectrum of the 2D SF-SIMM are shown in Fig 2. Obviously, the system is chaotic when *μ* ∈ (1.00, 1.65] ∪ (1.80, 2.64], and its complexity values are large. The system has two periodic windows at *μ* ∈ (1.65, 1.80] ∪ (2.64, 2.88], and its complexity values are relatively low.

**Fig 3:**
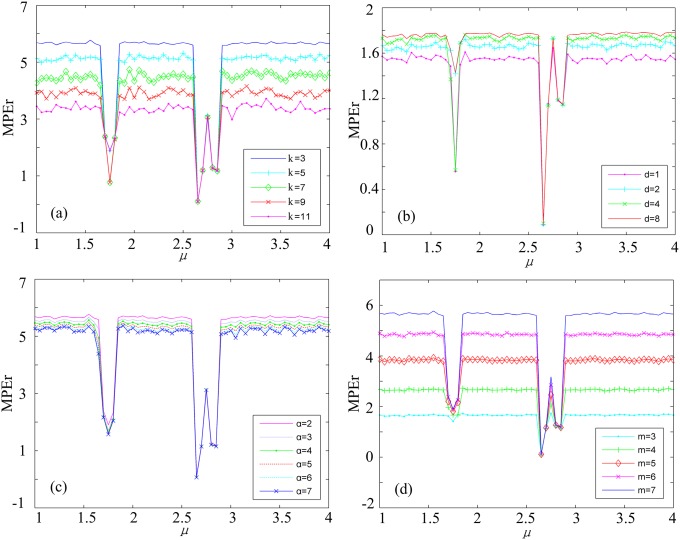
MPEr complexities with different parameters. (a) with different *k*. (b) with different *d*. (c) with different *α*. (d) with different *m*.

We choose the parameters by observing the variation of complexities with the dynamic characteristics of chaotic signals. The MPEr complexities with different parameters are shown in Fig 3. When *k* decreases, the differences of MPEr values between chaotic state and the periodic state are larger as shown in Fig 3(a). The same treads are detected when *α* decreases, and *m* increases as shown in Fig 3(c) and 3(d) respectively. Thus, we set *k* = 3, *α* = 2 and *m* = 7. To analyze the weighted factors, we introduce a parameter *d*, and it is the distance between neighbor weighted factors (*d* = |*w*_*n*_ − *w*_*n*−1_|). Setting the first weighted factor *w*_1_ = 1 (In fact, no matter what the first item is, the effect is the same when *d* is determined according to our numerical simulations), we obtain that the MPEr values with lager *d* are more sensitive to the differences of MPEr values between chaotic state and the periodic state as shown in Fig 3(b). Although the complexity value will be a little larger, when the parameter *d* increases, considering to balance the performance of the algorithm and the calculation time, *d* = 2 is the suitable choice. The weighting-averaging method emphasizes the central information (Here, *k* = 3, so there are 3 weighted factors, *w*_1_, *w*_2_, *w*_3_. *w*_2_ is used to emphasizes the central information). It means the weighted factors increase first, and then decrease. So, *w*_1_ = 1, *w*_2_ = *w*_1_ + *d* = 3, *w*_3_ = *w*_2_ − *d* = 1. Theoretically, the new parameters would lead to increase of the computational complexity of the algorithm. However, during numerical simulations, the difference of average calculation time between PEr and MPEr is less than one second. So far, all the parameters of the MPEr are determined by numerical simulation analysis. Once the parameters are determined, MPEr can be used to measure different EEG data according to our experiments results. So it is reasonable for practical applications.

**Fig 4:**
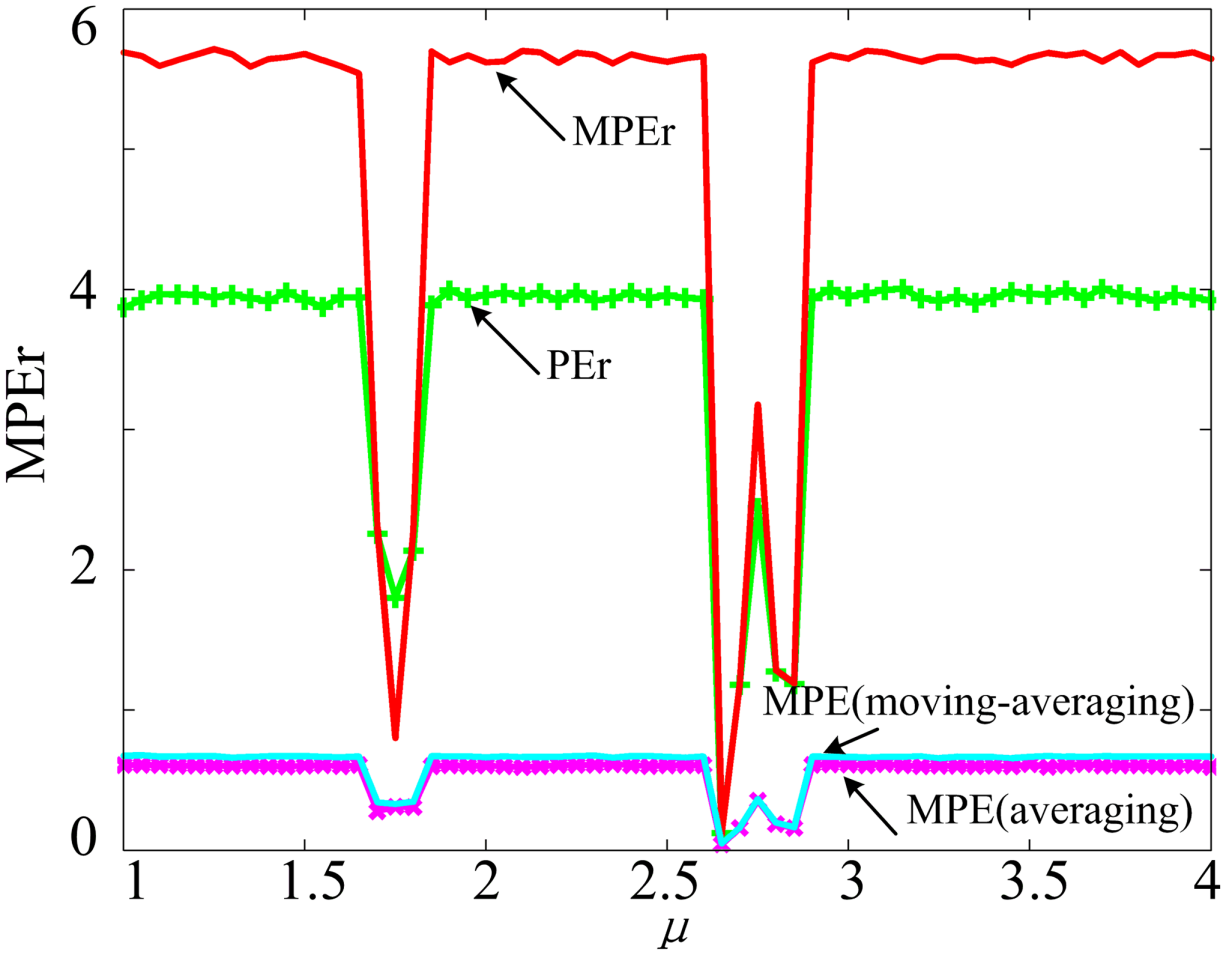
Complexities of MPEr (*m* = 7, *α* = 2, *w*_1_ = 1, *w*_2_ = 3, *w*_3_ = 1), PEr (*m* = 7, *α* = 2), PE with average method (*m* = 4, *s* = 4) and PE with moving-averaging method (*m* = 4, *s* = 4).

To show the performance of MPEr algorithm, the complexities of 2D SF-SIMM are analyzed by MPEr, PEr and MPE algorithms, respectively. We choose optimal parameters for the three algorithms by numerical simulations. As shown in Fig 4, MPEr and PEr can distinguish different dynamic states better. Furthermore, the MPEr values are larger than the values of PE with average and moving-averaging method when the system is chaotic. Thus the performance of MPEr is the best among that of these complexity measure algorithms, and the MPEr algorithm is applied to analyze EEG signals in next section.

## Results and discussion

### EEG data descriptions

**Fig 5:**
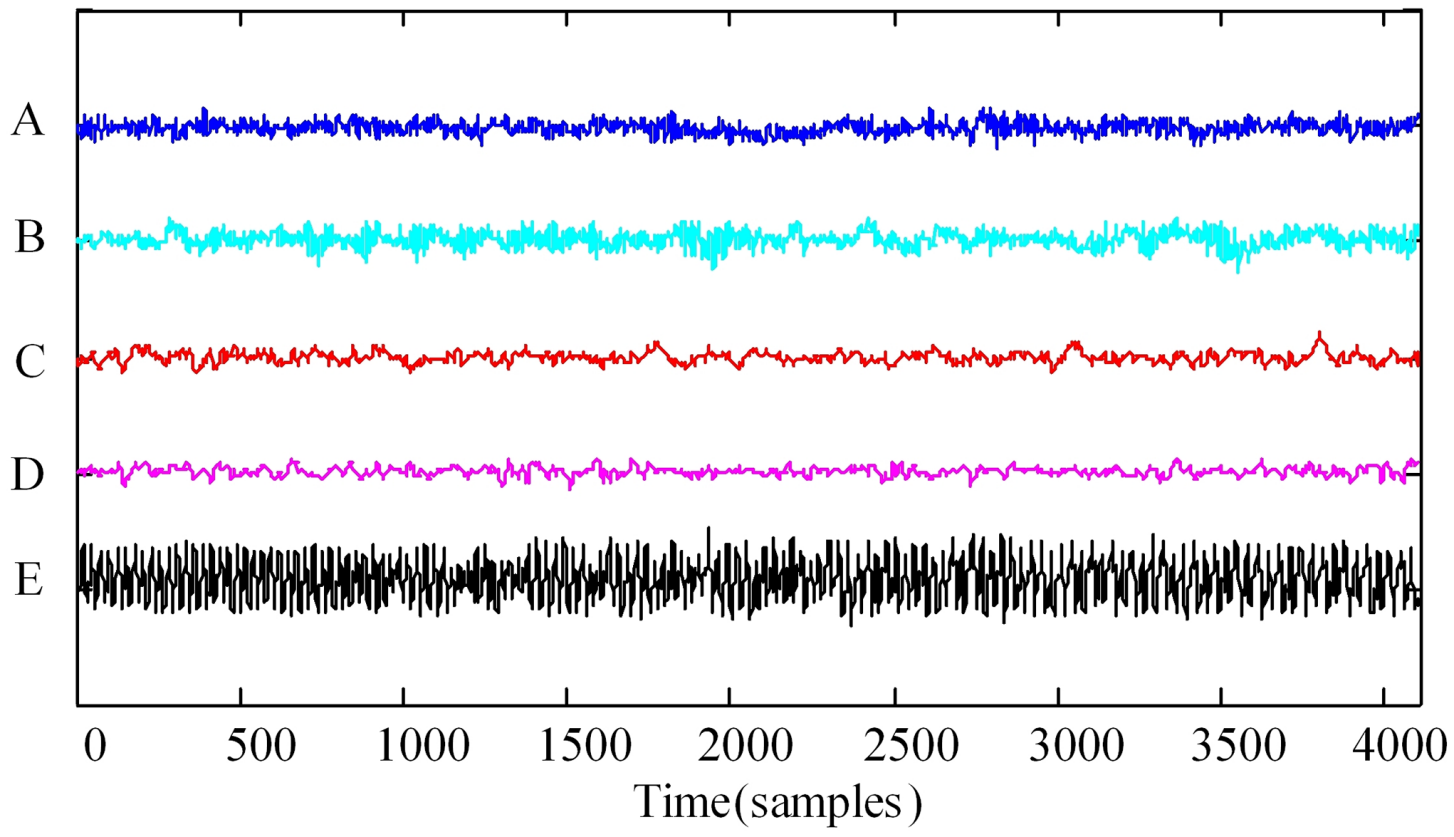
The time-domain waveforms of the 5-group EEG signals.

We examine 5-group (denoted with A-E) EEG recordings downloaded from the public database [42]. Each group of EEG recordings contain 100 single segments. The descriptions of EEG data are summarized in Table 1. Segments of groups A and B are taken from the depicted electrodes of healthy volunteers. Segments of groups C, D and E are taken from the depicted electrodes of epileptogenic zone of patients. All the EEG signals were recorded by the same 128-channel amplifier system. After 12-bit analog-to-digital conversion, the data were written continuously onto the disk of a data acquisition computer system at a sampling rate of 173.61 Hz. The duration of each segment is about 23.6 seconds and contains 4096 samples. The band width of the band-pass filter is 0.53-40 Hz. We plotted the time-domain waveforms of the 5-group EEG signals as shown in Fig 5.

**Table 1:** The EEG data.

Groups	Recordings
*A*	Healthy volunteers in an awake state with eyes open
*B*	Healthy volunteers in an awake state with eyes closed
*C*	Patients during seizure free intervals (the hemisphere of epileptogenic zone)
*D*	Patients during seizure free intervals (the opposite hemisphere of the epileptogenic zone)
*E*	Patients during seizure activity

### Complexity analysis of the EEG signals

**Fig 6:**
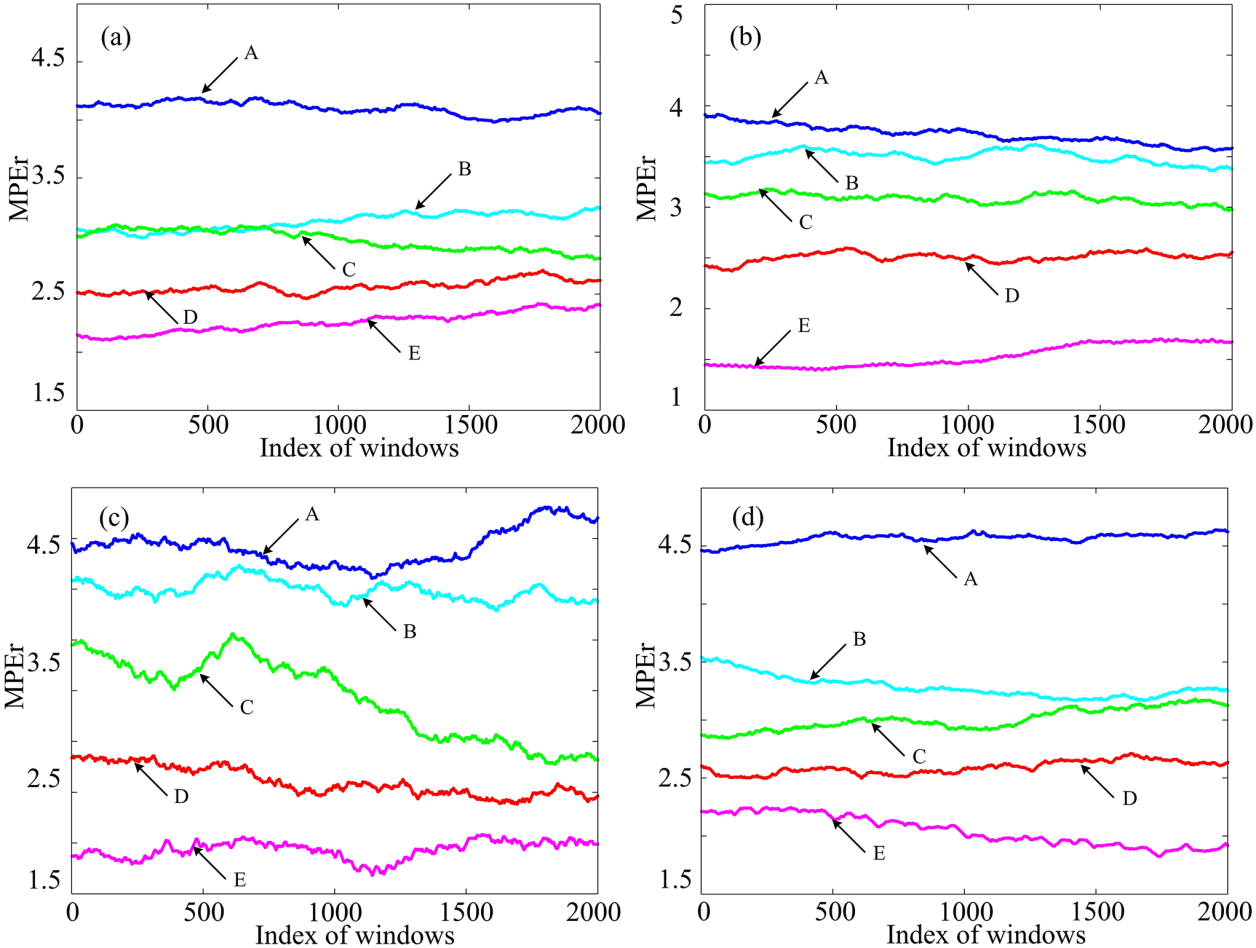
The MPEr complexities with 2000-width sliding window for different segments. (a) for segment 004. (b) for segment 008. (c) for segment 028. (d) for segment 029.

According to the procedure described above, the optimal parameter configurations for MPEr are determined. Now, we applied MPEr to the EEG signals by using sliding window method. The basic idea of sliding window is to create a *W*-width sliding window and count the sample points within the sliding window. Here, the width of the window *W* is 2000, and the sliding step size is 1. To show the performance of MPEr, we randomly choose 4 segments (004, 008, 028, 029) of every group of EEG signals and calculate the MPEr complexities as shown in Fig 6. Obviously, the MPEr complexity value of the healthy EEG signal is higher than that of the epileptic EEG signal. It is consistent with the results that the complexity of healthy EEG signal is higher than that of pathologic EEG signal [43, 44]. Moreover, the MPEr values decrease gradually from group A to group E. In fact, we have analyzed most of the 100 segments and the similar results are obtained. Thus, the MPEr algorithm has good discrimination performance.

### Boxplot analysis for the complexity of the EEG signals

**Fig 7:**
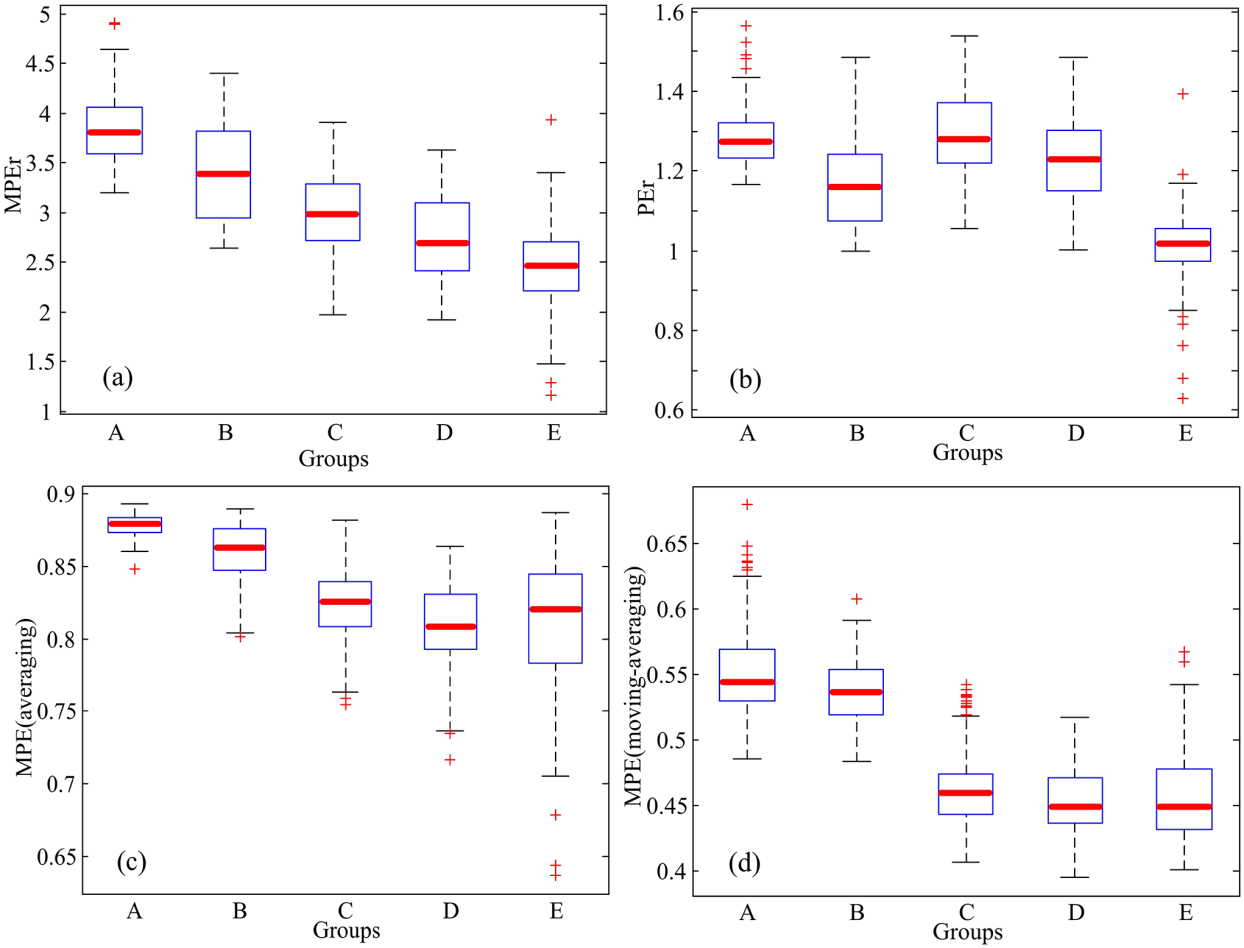
Boxplots of MPEr, PEr and MPE. (a) MPEr: *α* = 2, *m* = 7. (b) PEr: *α* = 2, *m* = 7. (c) MPE (average): *s* = 4, *m* = 4. (d) MPE (moving-averaging): *s* = 4, *m* = 4.

Boxplot is a method for graphically depicting groups of numerical data through their quartiles in statistics. The bottom and top of the box are the first and third quartiles, and the line inside the box is the median. The individual points are outliers. Boxplot is non-parametric and it can reflect dispersion of data and identify outliers [45]. The median in the box is shown by the bold line, which extends from 25% to 75%. The plus sign represents the outlier. To illustrate the discrimination performance better, all EEG segments in the database are analyzed and the boxplots of MPEr, PEr, MPE (average) and MPE (moving-averaging) are plotted as shown in Fig 7. MPEr complexities are higher than that of PEr and MPE (average and moving-averaging). MPEr has different mean values for all 5 groups, and the mean values are gradually decreased from group A to group E. While PEr results overlap with each other, and the mean values are not decreased gradually. Furthermore, there are more outliers in PEr than that in MPEr, which indicates that MPEr is more effective than PEr. For MPE (averaging), the mean values are gradually decreased from group A to group D, but the mean value of group E is greater than that of group D. For MPE (moving-averaging), the mean values of group A and group B are greater than those of group C, group D, and group E. However, the mean values of group A and group B almost overlap, so do that of group C, group D, and group E. So, MPEr can discriminate the EEG signals of healthy activity, epileptic seizure free intervals, and epileptic seizures intervals correctly.

### ANOVA and Dunnett-T3 analysis for the complexity of EEG signals

To validate the effectiveness of the complexity measures, one-way analysis of variance (ANOVA) is carried out. The essence of ANOVA is to judge whether there is a significant difference among all groups. In practice, ANOVA is used to estimate the significant influence of various factors on a dependent variable. Here, the dependent variable is MPEr complexity values, and the factor is the ‘Group’. The null hypothesis is that the 5-group EEG signals have the same MPEr complexity values. Setting *p* = 0.05 as the level of statistical significance for the test, we employ ANOVA to analyze the complexities and the results are listed in Table 2. Obviously, for MPEr, *F*(4,495) = 158.22, *p* = 0. The *p* value of ANOVA is less than 0.05, so the null hypothesis is irrational. That means all the MPEr complexity values of 5-group EEG signals are statistically significant.

**Table 2:** ANOVA for MPEr.

Algorithm		Sum of mean squares	df	Mean squares	*F*	*p*
*MPEr*	Between Groups	122.438	4	30.610	158.822	0.000
*MPEr*	Within Groups	95.401	495	0.193	-	-
*MPEr*	Total	217.839	499	-	-	-

To demonstrate the discrimination performance further, we carry out Dunnett-T3 test. Taking *p* = 0.05 as the level of statistical significance, and we obtained the results as shown in Table 3. *p* < 0.05 indicates significant differences and df means degree of freedom. For MPEr of each two groups, *p* = 0, which is less than 0.05. It means the MPEr complexities of different groups EEG signals are all statistically significant, so MPEr algorithm can discriminate 5-group EEG signals from each other.

**Table 3:** *p* values of Dunnett-T3 for MPEr.

**Groups**	**A**	**B**	**C**	**D**	**E**
*A*	*N*	1.912 × 10^−11^	1.776 × 10^−15^	4.441 × 10^−16^	1.443 × 10^−15^
*B*	1.912 × 10^−11^	*N*	4.812 × 10^−8^	0.000	0.000
*C*	1.776 × 10^−15^	4.812 × 10^−8^	*N*	2.160 × 10^−4^	1.902 × 10^−13^
*D*	4.441 × 10^−16^	0.000	2.160 × 10^−4^	*N*	2.611 × 10^−4^
*E*	1.443 × 10^−15^	0.000	1.902 × 10^−13^	2.611 × 10^−4^	*N*

To compare the performances of different complexity measure algorithms, ANOVA and Dunnett-T3 analysis are conducted. The performance of the MPEr algorithm is compared with PEr [24], MPE (averaging) [29], MPE (moving-averaging) [39] and weighted-permutation entropy (WPE) [46]. Taking *p* = 0.05 as the level of statistical significance for the tests. As shown in Table 4, the differences of MPEr complexities among 5 groups are more significant than that of PEr (MPEr (*F*(4,495) = 158.22, *p* < 0.05) > PEr (*F*(4,495) = 138.391, *p* < 0.05)). Furthermore, from Table 5, *p* value of PEr between group A and group C is 1.000, which is greater than 0.05. It means PEr complexities of these two groups are at the same level, so it fails to discriminate healthy EEG group A and seizure free EEG group C. MPEr (*F*(4,495) = 158.22, *p* < 0.05) > MPE(averaging) (*F*(4,495) = 92.299, *p* < 0.05). Moreover, Table 6 suggests that *p* value of MPE (averaging) between group C and group E is 0.316, which means MPE (averaging) complexities of these two groups are at the same level. So is that of group D and group E. Although MPEr (*F*(4,495) = 158.22, *p* < 0.05) < MPE (moving-averaging) (*F*(4,495) = 218.483, *p* < 0.05), the *p* value of MPE (moving-averaging) between group C and group E is 0.592, which is greater than 0.05. So are that of group C and group D, group D and group E as shown in Table 7. MPEr (*F*(4,495) = 158.22, *p* < 0.05) > WPE (*F*(4,495) = 50.167, *p* < 0.05) as shown in Tables 4 and 8 shows that *p* value of WPE between group C and group D is 0.188, which is greater than 0.05, so WPE fails to discriminate group C and group D.

**Table 4:** ANOVA for PEr, MPE (averaging) and MPE (moving-averaging).

Algorithms		Sum of mean squares	df	Mean squares	*F*	*p*
PEr [24]	Between Groups	5.588	4	1.397	138.391	0.000
PEr	Within Groups	4.997	495	0.010	-	-
PEr	Total	10.585	499	-	-	-
MPE [29]	Between Groups	0.370	4	0.092	92.299	0.000
MPE	Within Groups	0.496	495	0.001	-	-
MPE	Total	0.865	499	-	-	-
MPE [39]	Between Groups	0.903	4	0.226	218.483	0.000
MPE	Within Groups	0.511	495	0.001	-	-
MPE	Total	1.414	499	-	-	-
WPE [46]	Between Groups	4.109 × 10^−11^	4	1.027 × 10^−11^	50.167	1.900 × 10^−35^
WPE	Within Groups	1.014 × 10^−10^	495	2.408 × 10^−13^	-	-
WPE	Total	1.425 × 10^−10^	499	-	-	-

**Table 5:** *p* values of Dunnett-T3 for PEr.

**Groups**	**A**	**B**	**C**	**D**	**E**
*A*	*N*	1.329 × 10^−15^	1.000	3.665 × 10^−14^	0.000
*B*	1.329 × 10^−15^	*N*	2.232 × 10^−14^	0.002	2.492 × 10^−15^
*C*	1.000	2.232 × 10^−14^	*N*	0.001	0.000
*D*	3.665 × 10^−14^	0.002	0.001	*N*	1.564 × 10^−15^
*E*	0.000	2.492 × 10^−15^	0.000	1.564 × 10^−15^	*N*

**Table 6:** *p* values of Dunnett-T3 for MPE(averaging).

**Groups**	**A**	**B**	**C**	**D**	**E**
*A*	*N*	8.683 × 10^−13^	0.000	1.665 × 10^−15^	2.776 × 10^−15^
*B*	8.683 × 10^−13^	*N*	0.000	0.000	2.537 × 10^−12^
*C*	0.000	0.000	*N*	0.004	0.316
*D*	1.665 × 10^−15^	0.000	0.004	*N*	1.000
*E*	2.776 × 10^−15^	2.537 × 10^−12^	0.316	1.000	*N*

**Table 7:** *p* values of Dunnett-T3 for MPE(moving-averaging).

**Groups**	**A**	**B**	**C**	**D**	**E**
*A*	*N*	0.010	5.995 × 10^−15^	0.000	0.000
*B*	0.010	*N*	0.000	4.552 × 10^−15^	1.109 × 10^−15^
*C*	5.995 × 10^−15^	0.000	*N*	0.065	0.592
*D*	0.000	4.552 × 10^−15^	0.065	*N*	0.999
*E*	0.000	1.109 × 10^−15^	0.592	0.999	*N*

**Table 8:** *p* values of Dunnett-T3 for WPE.

**Groups**	**A**	**B**	**C**	**D**	**E**
*A*	*N*	1.110 × 10^−16^	9.766 × 10^−7^	5.590 × 10^−4^	0.000
*B*	1.110 × 10^−16^	*N*	1.364 × 10^−11^	8.689 × 10^−13^	0.000
*C*	9.766 × 10^−7^	1.364 × 10^−11^	*N*	0.188	1.051 × 10^−13^
*D*	5.590 × 10^−4^	8.689 × 10^−13^	0.188	*N*	8.882 × 10^−16^
*E*	0.000	0.000	1.051 × 10^−13^	8.882 × 10^−16^	*N*

Table 9 shows the comparison results of other methods by using statistical analysis. Here, *p* values of all methods are less than 0.05, and the *F* values of different methods are different. The *F* value of WPE is the smallest. For MPE (averaging), the *F* value is larger than that of WPE, and it cannot discriminate seizure free EEG group C, group D and seizure EEG group E. Although *F* value of MPE (moving-averaging) is the largest, it also fails to discriminate seizure free EEG group C, group D and seizure EEG group E. PEr also cannot discriminate healthy group A and seizure free group C. MPEr has higher *F* value and can discriminate 5-group EEG signals successfully. The statistical analysis results are consistent with that of the boxplot analysis. So, MPEr has better performance for analyzing epileptic EEG signals.

**Table 9:** Results by statistical analysis.

Algorithms	*F*	comparisons
*PEr*	138.391	Fail to discriminate group A and group C
*MPE*(*averaging*)	92.299	Fail to discriminate group C and group E,group D and group E
*MPE*(*moving*–*averaging*)	218.483	Fail to discriminate group C and group D,group C and group E,group D and group E
*WPE*	50.167	Fail to discriminate group C and group D
*MPEr*	158.822	Discriminate five groups successfully

## Conclusion

In this paper, a novel complexity measure algorithm, named multiscale permutation Rényi entropy (MPEr), is proposed by introducing weighting-averaging method. The complexity analysis of 2D SF-SIMM shows that MPEr has higher complexities than PEr and MPE, and it indicates that MPEr can discriminate different dynamic states better than other complexity measure algorithms. Furthermore, the suitable parameters are determined for MPEr by analyzing chaotic signals. We apply this new algorithm to analyze five groups of EEG recordings. We found that the complexity values of the healthy EEG signals are higher, while that of the epileptic EEG signals are lower. MPEr can distinguish different EEG signals effectively. The statistical analysis also supports that MPEr algorithm has better performance than that of other complexity measure algorithms. It provides prospect of further study in electrical activity of brain and may apply for clinical medical application in the future.
